# Paternal obesity modifies the effect of an antenatal lifestyle intervention in women who are overweight or obese on newborn anthropometry

**DOI:** 10.1038/s41598-017-01672-w

**Published:** 2017-05-08

**Authors:** Jodie M. Dodd, Lodewyk E. Du Plessis, Andrea R. Deussen, Rosalie M. Grivell, Lisa N. Yelland, Jennie Louise, Andrew J. Mcphee, Jeffrey S. Robinson, Julie A. Owens

**Affiliations:** 1 The University of Adelaide, Discipline of Obstetrics & Gynaecology, and Robinson Research Institute, Adelaide, South Australia Australia; 2 Department of Perinatal Medicine, Women’s and Children’s Hospital, North Adelaide, South Australia Australia; 3 South Australian Health and Medical Research Institute (SAHMRI), Adelaide, South Australia Australia; 4 The University of Adelaide, School of Public Health, Adelaide, South Australia Australia; 5 Department of Neonatal Medicine, Women’s and Children’s Hospital, North Adelaide, South Australia Australia; 6 Flinders University, Department of Obstetrics & Gynaecology, Bedford Park, South Australia Australia

## Abstract

The contribution of paternal obesity to pregnancy outcomes has been little described. Our aims were to determine whether the effect of an antenatal maternal dietary and lifestyle intervention among women who are overweight or obese on newborn adiposity, was modified by paternal obesity. We conducted a secondary analysis of a multicenter randomised trial. Pregnant women with BMI ≥25 kg/m^2^ received either Lifestyle Advice or Standard Care. Paternal anthropometric measures included height, weight, BMI; waist, hip, calf and mid-upper arm circumferences; biceps and calf skinfold thickness measurements (SFTM); and percentage body fat. Newborn anthropometric outcomes included length; weight; head, arm, abdominal, and chest circumferences; biceps, triceps, subscapular, suprailiac, thigh, and lateral abdominal wall SFTM; and percentage body fat. The effect of an antenatal maternal dietary and lifestyle intervention among women who were overweight or obese on neonatal anthropometric measures, was significantly modified by paternal BMI ≥35.0 kg/m^2^, with a significantly smaller infant triceps, suprailiac, and thigh SFTM, and percent fat mass, compared with that observed in offspring of lean fathers. Further research is required to determine whether our observed associations are causal, and whether paternal weight loss prior to conception is a potential strategy to reduce the intergenerational effects of obesity.

## Introduction

Globally 2.1 billion adults are overweight or obese^1^. In many developed countries, overweight and obesity has increased over the past three decades^1^, with over 70% of adult males and 60% of females having a body mass index (BMI) above 25.0 kg/m^2^ ^1^. Obesity contributes significantly to overall burden of disease^2^, being associated with increased risks of cardiovascular disease, type-2 diabetes and many malignancies^3^, contributing indirectly to more than 3.4 million deaths annually^4^.

Overweight and obesity during pregnancy represents a significant health burden, with almost 50% of women having a BMI above 25 kg/m^2^ on entering pregnancy^5^. Adverse effects of high maternal BMI on maternal pregnancy outcomes are well documented, the risks increasing as BMI increases^6, 7^. Furthermore, maternal obesity is associated with an increased risk of high infant birth weight, nursery admission, preterm birth, congenital anomalies, and both jaundice and hypoglycaemia^7^, with both high maternal BMI and gestational weight gain significant predictors of offspring obesity^8^. In an attempt to improve maternal and infant outcomes, interest has focused on interventions in the antenatal period to limit gestational weight gain, with systematic reviews and meta-analyses of randomized trials identifying only a modest effect on maternal weight change^9, 10^.

Paternal contributions to fetal growth, infant birth weight and body composition are less well described. Paternal height has been consistently correlated with birth weight and measures of skeletal growth, primarily birth length^11–15^. While some report positive associations between paternal weight and infant birth weight^16, 17^, this is not universal^14^. Furthermore, while some have identified associations between paternal BMI and infant birth weight^16, 18, 19^, others have not^11, 13, 20^. These studies have limitations. Most have not evaluated the contribution of paternal BMI to newborn anthropometric measures of adiposity, have involved relatively lean individuals, and have not adequately controlled for maternal factors contributing to fetal growth, or have relied upon maternal reporting of paternal weight and height.

The impact of paternal obesity on the efficacy of pregnancy interventions to limit gestational weight gain, particularly among overweight or obese women, has not been examined. We have reported the primary findings of the LIMIT randomised trial evaluating provision of antenatal dietary and lifestyle advice to women who were overweight or obese, indicating an 18% relative risk reduction in infant birth weight above 4 kg^21^, following improvements in maternal diet and physical activity^22^. The planned secondary study reported here, evaluated whether the effect of a randomised antenatal maternal dietary and lifestyle intervention among women who are overweight or obese on newborn adiposity measures, was modified by paternal BMI.

## Results

**Figure 1:**
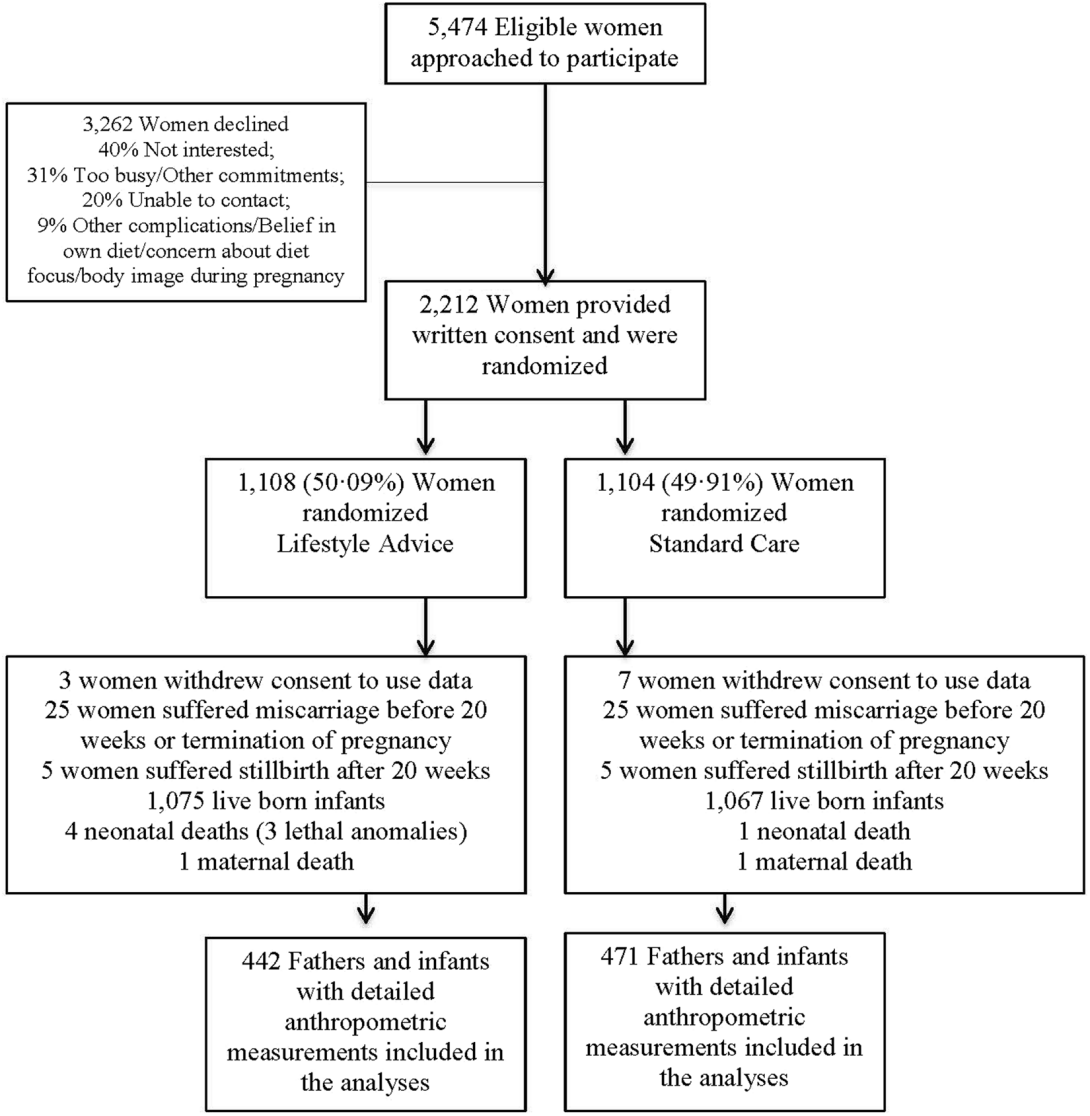
Flow of participants through the trial.

We randomized 2,212 women, (1,108 Lifestyle Advice; 1,104 Standard Care). There were 2,142 live-born infants (1,075 Lifestyle Advice; 1,067 Standard Care), with detailed anthropometric measurements for 913 fathers and infants (442 Lifestyle Advice; 471 Standard Care) (Fig. 1). Baseline characteristics of mothers of these infants were similar between treatment groups (Table 1), and similar to the full randomised groups^21^. Paternal (Table 2) and neonatal^23^ anthropometric measurements were also similar between treatment groups. There was a statistically significant correlation between maternal and paternal BMI (Pearson correlation coefficient 0.22, p < 0.001); a higher proportion of fathers were in the obese category for the highest maternal BMI groups compared to the lowest; however more than 50% of fathers had BMI <30 in every maternal BMI category.

**Table 1:** Maternal baseline characteristics by randomised group.

Characteristic	Lifestyle Advice (N = 442)	Standard Care (N = 471)
Maternal Age (Years)*	29.5 (5.7)	29.6 (5.4)
Gestational Age at Entry (Weeks)^+^	14.0 (11.9–16.6)	14.1 (11.7–16.7)
Body Mass Index (kg/m^2^)^+^	31.1 (27.9–36.2)	31.3 (27.6–36.3)
Body Mass Index Category^#^		
BMI 25.0–29.9	187 (42.3)	190 (40.3)
BMI 30.0–34.9	112 (25.3)	135 (28.7)
BMI 35.0–39.9	90 (20.4)	79 (16.8)
BMI >=40.0	53 (12.0)	67 (14.2)
Public Patient^#^	429 (97.1)	461 (97.9)
Caucasian^#^	412 (93.2)	431 (91.5)
Smoker^#^	41 (9.3)	48 (10.2)
Nulliparous^#^	210 (47.5)	231 (49.0)
Index of Socio-economic Disadvantage^		
Quintile 1 (Most Disadvantaged)	123 (27.8)	130 (27.6)
Quintile 2	115 (26.0)	135 (28.7)
Quintile 3	69 (15.6)	67 (14.2)
Quintile 4	64 (14.5)	74 (15.7)
Quintile 5 (Least Disadvantaged)	71 (16.1)	65 (13.8)

*Mean and standard deviation. ^+^Median and interquartile range. ^**#**^Number and %. ^Socioeconomic index as measured by SEIFA.

**Table 2:** Paternal anthropometric characteristics by maternal randomised treatment group.

Characteristic	Lifestyle Advice (N = 442)	Standard Care (N = 471)
Weight (kg)*	91.9 (18.4)	91.9 (18.7)
Height (cm)*	179.0 (6.8)	179.1 (7.5)
Body Mass Index (kg/m^2^)*	28.7 (5.6)	28.6 (5.4)
Body Mass Index Category^#^		
Missing	8 (1.8)	10 (2.1)
BMI <25	110 (24.9)	110 (23.4)
BMI 25-<30	183 (41.4)	193 (41.0)
BMI 30-<35	89 (20.1)	110 (23.4)
BMI >=35	52 (11.8)	48 (10.2)
Age (years)*,^	32.58 (6.34)	32.54 (6.35)
Calculated Fat Mass (kg)^+^	17.2 (11.3–24.8)	17.7 (11.8–24.8)
Calculated Percent Body Fat^+^	19.4 (14.3–25.0)	19.3 (14.4–25.2)

*Mean and standard deviation. ^+^Median and interquartile range. ^#^Number and %. ^Based on a subset of 197 fathers (101 Lifestyle Advice, 96 Standard Care) for whom information on date of birth was available.

### Modification of the antenatal dietary and lifestyle intervention on neonatal anthropometry by paternal BMI

Increasing paternal BMI significantly modified the effect of the antenatal intervention on infant triceps (p = 0.03) and suprailiac (p = 0.02) skinfold thickness measurement (SFTM), with some evidence to suggest paternal BMI was an effect modifier for thigh SFTM (p = 0.06) (Table 3). Specifically, in infants born to a father with BMI ≥35.0 kg/m^2^ the antenatal intervention was associated with smaller average triceps SFTM by 0.89 mm while no significant effect of the intervention was seen for infants born to fathers with BMI <25.0 kg/m^2^, or 30.0–34.9 kg/m^2^. Paternal BMI ≥35.0 kg/m^2^ modified the effect of the intervention for average infant suprailiac SFTM, which was smaller by 1.87 mm in the intervention group, in contrast to infants of fathers with BMI <25.0 kg/m^2^, 25.0–29.9 kg/m^2^ and 30.0–34.9 kg/m^2^ respectively.

**Table 3:** Treatment effect modification of infant morphology, according to paternal body mass index category.

Infant Outcome	Paternal BMI Category	Lifestyle Advice (N = 442)	Standard Care (N = 471)	Adjusted Treatment Effect (95% CI)	Adjusted P-value	Adjusted Interaction P-value
Birth Weight (g)	BMI <25	3470.45 (473.04)	3492.44 (547.94)	−26.19 (−167.95, 115.57)	0.712	0.93
	BMI 25–<30	3555.73 (466.05)	3520.90 (547.15)	22.06 (−86.62, 130.74)	0.69	
	BMI 30–<35	3515.25 (546.53)	3512.65 (708.76)	−20.99 (−169.61, 127.63)	0.78	
	BMI >=35	3538.54 (491.83)	3560.77 (509.84)	−34.80 (−244.12, 174.52)	0.75	
Birth Length (cm)	BMI <25	49.94 (2.14)	50.00 (2.37)	−0.08 (−0.71, 0.55)	0.8	0.64
	BMI 25–<30	50.18 (1.90)	50.09 (2.31)	0.06 (−0.42, 0.54)	0.79	
	BMI 30–<35	50.01 (2.31)	49.59 (3.24)	0.38 (−0.28, 1.03)	0.26	
	BMI >=35	49.64 (2.30)	49.84 (2.13)	−0.31 (−1.24, 0.62)	0.51	
Head Circumference (cm)	BMI <25	34.94 (1.29)	34.98 (1.70)	−0.10 (−0.52, 0.33)	0.66	0.41
	BMI 25–<30	34.91 (1.41)	34.88 (1.64)	0.03 (−0.29, 0.36)	0.84	
	BMI 30–<35	34.92 (1.42)	34.63 (2.01)	0.24 (−0.21, 0.68)	0.29	
	BMI >=35	34.66 (1.78)	35.06 (1.71)	−0.40 (−1.04, 0.23)	0.21	
Chest Circumference (cm)	BMI <25	34.23 (1.78)	34.45 (1.91)	−0.25 (−0.93, 0.44)	0.48	0.4
	BMI 25–<30	34.39 (1.79)	34.09 (2.02)	0.35 (−0.18, 0.89)	0.2	
	BMI 30–<35	34.27 (2.12)	34.45 (2.12)	−0.32 (−1.04, 0.40)	0.39	
	BMI >=35	33.92 (1.87)	33.86 (2.35)	−0.15 (−1.11, 0.82)	0.76	
Mid Upper Arm Circumference (cm)	BMI <25	11.29 (0.89)	11.19 (1.18)	0.02 (−0.34, 0.38)	0.91	0.82
	BMI 25–<30	11.30 (1.01)	11.18 (1.11)	0.10 (−0.18, 0.38)	0.49	
	BMI 30–<35	11.22 (0.90)	11.12 (0.98)	0.03 (−0.35, 0.41)	0.88	
	BMI >=35	11.23 (1.05)	10.90 (1.08)	0.30 (−0.21, 0.81)	0.25	
Abdominal Circumference (cm)	BMI <25	32.95 (2.10)	32.82 (2.35)	−0.02 (−0.79, 0.75)	0.95	0.18
	BMI 25–<30	32.99 (2.13)	32.59 (2.34)	0.40 (−0.20, 1.00)	0.2	
	BMI 30–<35	32.72 (2.23)	33.15 (2.24)	−0.67 (−1.48, 0.13)	0.1	
	BMI >=35	32.69 (1.96)	32.26 (2.52)	0.40 (−0.68, 1.49)	0.47	
Biceps SFTM (mm)	BMI <25	4.23 (0.94)	4.13 (1.36)	0.07 (−0.34, 0.48)	0.74	0.81
	BMI 25–<30	4.60 (1.33)	4.41 (1.15)	0.16 (−0.16, 0.49)	0.32	
	BMI 30–<35	4.38 (1.26)	4.19 (1.02)	0.19 (−0.24, 0.63)	0.39	
	BMI >=35	4.20 (1.07)	4.35 (1.03)	−0.13 (−0.72, 0.45)	0.65	
**Triceps SFTM (mm)**	BMI <25	5.33 (1.27)	5.37 (1.57)	−0.13 (−0.61, 0.36)	0.61	**0.03**
	BMI 25–<30	5.61 (1.30)	5.32 (1.44)	0.27 (−0.11, 0.65)	0.16	
	BMI 30–<35	5.25 (1.48)	5.33 (1.29)	−0.14 (−0.64, 0.37)	0.59	
	**BMI** >=**35**	**5.03 (1.12)**	**5.94 (1.50)**	−**0.89 (**−**1.57**, −**0.22)**	**0.01**	
**Suprailiac SFTM (mm)**	BMI <25	5.57 (1.76)	5.55 (1.67)	−0.09 (−0.75, 0.57)	0.79	**0.002**
	BMI 25–<30	5.94 (1.67)	5.73 (1.87)	0.21 (−0.30, 0.72)	0.42	
	BMI 30–<35	5.72 (2.20)	5.77 (2.11)	−0.08 (−0.77, 0.62)	0.83	
	**BMI** >=**35**	**5.12 (1.52)**	**7.03 (2.20)**	−**1.87 (**−**2.81**, −**0.93)**	**0.0001**	
Subscapular SFTM (mm)	BMI <25	5.25 (1.17)	5.05 (1.13)	0.14 (–0.32, 0.60)	0.55	0.64
	BMI 25–<30	5.13 (1.42)	5.08 (1.27)	0.06 (−0.30, 0.42)	0.75	
	BMI 30–<35	5.02 (1.49)	5.10 (1.16)	−0.14 (−0.62, 0.34)	0.56	
	BMI >=35	5.12 (1.19)	5.44 (1.64)	−0.31 (−0.96, 0.33)	0.34	
Lateral Abdominal SFTM (mm)	BMI <25	3.72 (1.05)	3.65 (1.03)	0.06 (−0.32, 0.43)	0.77	0.55
	BMI 25–<30	4.02 (1.21)	3.83 (1.04)	0.19 (−0.11, 0.49)	0.21	
	BMI 30–<35	3.84 (1.07)	3.66 (1.01)	0.13 (−0.27, 0.52)	0.53	
	BMI >=35	3.63 (0.92)	3.93 (0.88)	−0.26 (−0.79, 0.28)	0.35	
Thigh SFTM (mm)	BMI <25	6.68 (1.79)	6.54 (1.81)	0.06 (−0.60, 0.71)	0.86	0.06
	BMI 25–<30	7.40 (1.92)	6.99 (1.89)	0.45 (−0.06, 0.95)	0.08	
	BMI 30–<35	6.97 (2.21)	6.92 (1.72)	−0.06 (−0.74, 0.62)	0.86	
	BMI >=35	6.61 (1.65)	7.67 (2.14)	−1.01 (−1.92, −0.09)	0.03	
Calculated Fat Mass (g)	BMI <25	519.15 (161.25)	514.15 (169.81)	−0.37 (−67.79, 67.05)	0.99	0.12
	BMI 25–<30	546.39 (169.81)	508.34 (196.11)	37.26 (−14.61, 89.13)	0.16	
	BMI 30–<35	520.08 (239.72)	528.87 (197.73)	−20.21 (−89.36, 48.94)	0.57	
	BMI >=35	478.74 (137.34)	555.62 (200.03)	−89.49 (−182.90, 3.93)	0.06	
Calculated Fat Free Mass (g)*	BMI <25	3058.33 (282.29)	3062.06 (357.21)	−7.80 (−131.49, 115.90)	0.9	0.88
	BMI 25–<30	3069.61 (305.55)	3009.87 (360.21)	52.80 (−42.36, 147.97)	0.28	
	BMI 30–<35	3066.72 (380.31)	3027.67 (389.34)	5.19 (−121.68, 132.05)	0.94	
	BMI >=35	2986.76 (375.95)	2910.80 (380.12)	28.40 (−142.99, 199.78)	0.75	
**Calculated Percent Body Fat**	BMI <25	14.31 (3.39)	14.09 (3.29)	0.12 (−1.13, 1.38)	0.85	**0.02**
	BMI 25–<30	14.85 (3.28)	14.03 (3.61)	0.84 (−0.12, 1.81)	0.09	
	BMI 30–<35	13.93 (4.28)	14.50 (3.27)	−0.70 (−1.99, 0.59)	0.29	
	**BMI** >=**35**	**13.63 (2.78)**	**15.63 (3.77)**	−**2.15 (**−**3.89**, −**0.40)**	**0.02**	

Values are mean (SD) and treatment effects are differences in means.

Where the interaction p-value is not significant, treatment group comparisons within subgroups should be interpreted with caution.

Results have been adjusted for centre, maternal parity, maternal BMI, maternal age, socioeconomic status and maternal smoking status.

*Calculated using equation from Deierlein *et al*.^42^. Note that ‘ethnicity’ (Hispanic vs non-Hispanic) is included in this equation but is not applicable to the LIMIT cohort (all participants are ‘non-Hispanic’).

Paternal BMI ≥35.0 kg/m^2^ also positively modified the effect of the antenatal intervention on infant thigh SFTM, being associated with smaller average thigh SFTM of 1.01 mm, when compared with the small, non-significant treatment effects seen among infants of fathers with BMI <25.0 kg/m^2^, 25.0–29.9 kg/m^2^, and 30.0–34.9 kg/m^2^ respectively. This finding should be interpreted with caution however, as the treatment group by paternal BMI category interaction test did not reach statistical significance (p = 0.06).

Paternal BMI ≥35 kg/m^2^ also positively modified infant percentage body fat in response to the intervention (p = 0.02; Table 3). Treatment effects were again seen for infants of fathers in the highest BMI category only, where the intervention was associated with significantly smaller percent body fat by 2.15%.

Increasing paternal BMI did not significantly modify the effect of the antenatal intervention on mean infant birth weight, birth length, or body circumferences (Table 3).

In the Standard Care group only, paternal BMI category was associated with significant differences in mean suprailiac SFTM (p = 0.005) and thigh SFTM (p = 0.04), and there was some evidence of a difference in mean percent body fat (p = 0.06), but there were no other statistically significant differences between subgroups (Table 4).

**Table 4:** Associations between infant morphology and paternal body mass index category from participants in the Standard Care Group.

Infant Characteristic	Paternal BMI <25 (N = 110)	Paternal BMI 25-<30 (N = 193)	Paternal BMI 30-<35 (N = 110)	Paternal BMI >=35 (n = 48)	P value
Weight (g)	3492.44 (547.94)	3520.90 (547.15)	3512.65 (708.76)	3560.77 (509.84)	0.37
Length (cm)	50.00 (2.37)	50.09 (2.31)	49.59 (3.24)	49.84 (2.13)	0.84
Head Circumference (cm)	34.98 (1.70)	34.88 (1.64)	34.63 (2.01)	35.06 (1.71)	0.18
Chest Circumference (cm)	34.45 (1.91)	34.09 (2.02)	34.45 (2.12)	33.86 (2.35)	0.12
Mid Upper Arm Circumference (cm)	11.19 (1.18)	11.18 (1.11)	11.12 (0.98)	10.90 (1.08)	0.83
Abdominal Circumference (cm)	32.82 (2.35)	32.59 (2.34)	33.15 (2.24)	32.26 (2.52)	0.13
Biceps SFTM (mm)	4.13 (1.36)	4.41 (1.15)	4.19 (1.02)	4.35 (1.03)	0.45
Triceps SFTM (mm)	5.37 (1.57)	5.32 (1.44)	5.33 (1.29)	5.94 (1.50)	0.07
Suprailiac SFTM (mm)	5.55 (1.67)	5.73 (1.87)	5.77 (2.11)	7.03 (2.20)	**0.005**
Subscapular SFTM (mm)	5.05 (1.13)	5.08 (1.27)	5.10 (1.16)	5.44 (1.64)	0.15
Lateral Abdominal SFTM (mm)	3.65 (1.03)	3.83 (1.04)	3.66 (1.01)	3.93 (0.88)	0.51
Thigh SFTM (mm)	6.54 (1.81)	6.99 (1.89)	6.92 (1.72)	7.67 (2.14)	**0.04**
Fat Mass (g)	514.15 (169.81)	508.34 (196.11)	528.87 (197.73)	555.62 (200.03)	0.22
Fat Free Mass (g)	3062.06 (357.21)	3009.87 (360.21)	3027.67 (389.34)	2910.80 (380.12)	0.24
Percent Body Fat	14.09 (3.29)	14.03 (3.61)	14.50 (3.27)	15.63 (3.77)	0.06

Values are mean (SD).

*P-value from global test for effect of paternal BMI category. Results have been adjusted for maternal weight, height, BMI category, gestational weight gain, gestational diabetes, smoking status, socio-economic status, gestational age at birth and infant sex.

## Discussion

Our findings identify an association between high paternal BMI and some measures of newborn adiposity. Specifically, the effect of the antenatal intervention in terms of smaller anthropometric measures of adiposity was significantly enhanced in infants whose fathers had a BMI ≥35.0 kg/m^2^, as demonstrated by smaller average infant triceps, suprailiac, and thigh SFTM, and infant percent fat mass in the intervention group.

Strengths of our study include its prospective, randomised nature and rigorous methodology, utilising standardised and well-accepted research standard anthropometric measures, adhering to defined protocols^23, 24^. In analysing the effect of paternal BMI on infant adiposity, we have controlled for maternal BMI, gestational weight gain, and other pregnancy factors influencing fetal growth and therefore birth weight. Importantly, our study allows us to address the potential contribution of paternal obesity to a range of infant anthropometric measures that include adiposity, which has not been possible to date, as previously reported cohorts have utilised relatively lean populations, with few participants at the upper extremes of the BMI spectrum^11–16, 25^.

Limitations include lack of a gold standard measure of paternal and infant adiposity, however the use of dual x-ray absorptiometry or air displacement plethysmography was prohibitive in the context of this large randomised trial. We have previously reported good reproducibility with infant skin fold measurements^23^. Timing of partner measurements would ideally have been at the woman’s first pregnancy visit, however women were not enrolled until several weeks after this visit, and paternal measures were taken on average at about 90 days after randomisation. Average young adult male weight change in one year is 0.6 kg^26^, and over the five-month time frame in which paternal measurements were obtained were considered unlikely to change. We also conducted further analyses (not reported) to check for any effect of the intervention on paternal measures; these found no differences between the Lifestyle Advice and Standard Care groups.

The effects of paternal height on newborn length and measures of skeletal growth^11, 25^ and weight^11–16^, have been reasonably well described and appear to be independent of both maternal height and weight. There are well reported associations between paternal height and both newborn length or measures of skeletal growth^11, 25^, and weight^11–16^, across varying populations world-wide, and over different time periods. Catalano and colleagues^27^ reported a weak positive association between paternal height and estimated infant fat free mass, the outcome more strongly associated with infant sex, gestational age at birth, maternal gestational weight gain, and maternal BMI prior to conception.

The contribution of paternal weight and BMI to infant anthropometric measures has been less clearly established to date. While the majority of studies have failed to identify associations between either paternal weight or BMI and infant birth weight^11–15^, Mikulandra and colleagues^16^ report on almost 1,600 newborns from Croatia, identifying a significant positive association between increasing paternal weight and BMI, and increasing infant birth weight. These findings are consistent with those of Chen and colleagues in an Asian population^18^, and Klebanoff and colleagues, who utilized Danish population linked data from women birthing between 1974 and 1989^19^. In contrast, other studies conducted in predominantly Caucasian populations^11, 13, 20^ have not identified an association between increasing paternal BMI and infant birth weight. Most of these studies^11, 13, 16, 18–20^ have focused on infant birth weight, rather than investigating more direct measures of infant adiposity as we have.

The contribution of paternal obesity to human pregnancy outcomes has been previously poorly documented. The association between paternal BMI and infant adiposity described here may reflect paternal genetic influences, or paternal influences acting through the gamete environment prior to conception, or both. Our novel findings indicate that paternal obesity also significantly modifies the effect of an antenatal dietary and lifestyle intervention among pregnant women who are overweight or obese, on neonatal anthropometric measures of adiposity. One potential hypothesis to explain this observation may be that with increasing paternal BMI there is greater “familial” awareness of obesity, with women whose partners are of higher BMI being more supported to initiate changes to dietary and physical activity patterns. We investigated this possibility in a post-hoc analysis, but found no supporting evidence, with no substantial variation in maternal dietary and physical activity patterns across the paternal BMI spectrum.

There is increasing experimental evidence to support a male contribution to the development of offspring obesity, independent of genetics and a shared postnatal environment. Observational human literature identifies well recognized associations between paternal obesity, and an altered hormonal environment, impaired spermatogenesis, with reduced sperm counts^28, 29^ and impaired DNA integrity and mitochondrial function, secondary to increased reactive oxygen species^28^. Furthermore, these obesity related effects on spermatogenesis appear reversible through weight loss, either from dietary modification^28^, or following bariatric surgery^29^.

Animal based studies have identified similar reversible changes in spermatogenesis following diet-induced obesity in rodents^30, 31^, with suggestions that this impacts blastocyst function in early conception^32^. Furthermore, rodents fed a high-fat diet to induce obesity, both in the presence^33^ and absence^34^ of impaired glucose homeostasis, father offspring, particularly females, who are heavier, have reduced glucose tolerance, increased insulin resistance, and increased adiposity^33, 34^. Many of these adiposity effects persist in subsequent generations^35^.

While there is evidence to support a role for a male contribution to the development of offspring obesity, the precise mechanism whereby this “transmission” occurs remains uncertain^36^. Hypotheses include aberrant repair of spermatozoa DNA damage^37^, epigenetic changes reflecting hypo-methylation of histone bound DNA regions^33, 38^, and changes to non-coding small RNAs, such as micro RNAs^39^.

Of particular interest are the mechanisms underlying the effect of paternal BMI on the efficacy of the intervention in substantially ameliorating the increased infant adiposity associated with a high paternal BMI. The intervention, which improved maternal diet quality and physical activity^22^, presumably targets pathways in the fetus that mediate paternal genetic or preconception environmental influences on infant adiposity. These appear distinct from those harnessed by maternal obesity as by contrast, the intervention did not improve infant adiposity associated with a high maternal BMI^23^. Identifying the paternal pathways contributing to excess fetal adipose accumulation may reveal new targets and potential interventions to limit this.

Our findings highlight the need for ongoing research within human male populations into the impact of obesity on pregnancy and offspring health. Further research is required to determine whether our observed associations are causal, and, if so, whether paternal weight loss prior to conception would affect infant adiposity, as a potential strategy to reduce the adverse intergenerational effects of obesity.

## Subjects and Methods

### Study Design and Participants

The methodology^40^ and clinical findings have been reported^21, 22, 41^, and the trial registered on the Australian and New Zealand Clinical Trials Registry (ACTRN12607000161426). The LIMIT Study has been conducted in accordance with ethical and legal guidelines at the time the study was undertaken. Protocols used in this study were approved by the Women’s and Children’s Local Health Network Human Research and Ethics Committee at the Women’s and Children’s Hospital, the Central Northern Adelaide Health Service Ethics of Human Research Committee (Lyell McEwin Hospital) and the Flinders Clinical Research Ethics Committee (Flinders Medical Centre). Study methods complied with the Australian National Health and Medical Research Council’s National Statement on Ethical Conduct in Human Research (2007) - Updated May 2015 (https://www.nhmrc.gov.au/guidelines-publications/e72). Women with a singleton pregnancy between 10^+0^–20^+0^ weeks gestation, with BMI ≥25 kg/m^2^ were eligible.

### Intervention

At the first antenatal appointment, women had their height and weight measured, and BMI calculated. Eligible women were presented with written information prior to providing consent. The central randomisation service utilised a computer-generated schedule with balanced variable blocks, and stratification for parity (0 versus 1/more), BMI at booking (25–29.9 kg/m^2^ versus ≥30 kg/m^2^), and collaborating centre. Women were randomised to either ‘Lifestyle Advice’ or ‘Standard Care’.

#### Lifestyle Advice Group

Women participated in a comprehensive dietary and lifestyle intervention over pregnancy, delivered by a research dietician and research assistants^21, 22^.

#### Standard Care Group

Women continued their care according to local hospital guidelines, which did not include dietary, exercise, or gestational weight gain advice.

### Outcome Variables

#### Maternal BMI and gestational weight gain

At antenatal booking, women were categorised as overweight (BMI 25.0–29.9 kg/m^2^) or obese (BMI ≥30 kg/m^2^). Gestational weight gain was the difference in weight between 36 weeks gestation (or nearest to birth) and trial entry.

#### Paternal BMI and Anthropometry

Following informed consent, trained research assistants obtained detailed anthropometric measurements from the infant’s father during his partner’s pregnancy. Weight and height were measured to the nearest 0.1 kg and 0.1 cm respectively, in light clothing without shoes. BMI was categorised as normal (BMI <25 kg/m^2^), overweight (BMI 25.0–29.9 kg/m^2^), obese (BMI 30.0–34.9 kg/m^2^), or morbidly obese (BMI ≥35.0 kg/m^2^).

Waist, hip, calf, and mid-upper arm circumferences were obtained, and Harpenden skinfold callipers used to measure biceps and calf skinfold thicknesses. The following formula, derived from a demographically matched sample within an extensive database of complete anthropometric measurements, was used as a surrogate estimate of percentage body fat, total fat mass, and total non-fat mass (personal communication, Professor Timothy Olds, 17/10/12).

$$\begin{array}{rcl}BF & = & {4.3}+{0.099}\,\,Mass-{0.099}\,Ht+{0.564}\,\,BicepsSF-{0.076}\,ArmG\\  &  & +\,{0.539}\,\,CalfSF+{0.166}\,WaistG\end{array}$$

where BF = Body fat (%), Mass = Body Weight (kg), Ht = Height (cm), BicepsSF = Biceps skin fold thickness (mm), ArmG = Arm girth (cm), CalfSF = Calf skinfold thickness (mm), and WaistG = Waist Girth (cm).

#### Newborn Anthropometry

Prior to discharge from hospital, a trained research assistant obtained newborn anthropometric measures on healthy newborn infants on the postnatal ward, including birth weight, length, body circumference (head, arm, chest, and abdomen) and skinfold thickness measurements (SFTM) (biceps, triceps, subscapular, supra-iliac, thigh and lateral abdominal)^23^.

Infant gender, age in days and the sum of triceps, subscapular and thigh SFTM were used to calculate fat mass^42^. Percent body fat was calculated (fat mass/body weight at birth) × 100.

### Statistical Analyses

To evaluate whether the effect of a randomised antenatal maternal dietary and lifestyle intervention among women who are overweight or obese on newborn adiposity measures, was modified by paternal BMI, we conducted intention to treat analyses, according to the woman’s allocated treatment group at randomisation. Live born infants were included if their mother consented to anthropometric measurements being taken, and one or more paternal anthropometric measurements were available. Linear regression models were used to test whether the effect of treatment was modified by paternal BMI category and other measures of paternal adiposity, by including an intervention-by-paternal BMI category term in the model. Post-hoc tests were performed to estimate the effect of treatment group within each paternal BMI category and results are presented as differences in means with 95% CI. Adjustment was made for centre, parity, maternal BMI category, maternal age, socio-economic status and maternal smoking status.

To evaluate whether there was an association between paternal BMI and infant adiposity, analyses were conducted using data from the Standard Care group only. Linear regression models were used to test whether there was a difference in infant outcome by paternal BMI category (categories being used rather than continuous BMI to allow for nonlinearity of association and to aid interpretability). Separate estimates of difference in means were derived for each paternal BMI category. Analyses were adjusted for maternal BMI, gestational weight gain, gestational diabetes, socioeconomic status, gestational age at delivery, and maternal smoking.

Statistical significance was assessed at the 2-sided p < 0.05 level. As these are secondary analyses (with the findings considered exploratory), no adjustment for multiple comparisons has been made^43, 44^. All analyses were performed using SAS v9.3 (Cary, NC, USA).
